# Construct validity and reliability of Amharic version of DASS-21 scale among Ethiopian Defense University College of Health Science students

**DOI:** 10.1186/s12913-024-11267-7

**Published:** 2024-08-09

**Authors:** Bitew Sintayehu Tsegaye, Meseret Molla Asemu, Habtamu Belay Hailu

**Affiliations:** College of Health Sciences, Ethiopian Defense University, Bishoftu, Ethiopia

**Keywords:** DASS-21 scale, Validity, Reliability, University students, Ethiopia

## Abstract

**Background:**

DASS-21 is the common and widely used tool for assessing depression, anxiety and stress. However, its validity and Reliability in Ethiopian Amharic language is not assessed.

**Objective:**

To translate the DASS-21 and assess its validity and reliability among Ethiopian Defense University college of health science students in Bishoftu, Ethiopia.

**Method:**

An institutional based cross-sectional study was conducted using a self-administered questionnaire. A total of 435 students from different departments in accordance with their proportional size were participated in this study. As to the sampling technique, the study units were selected from each department and year of study using simple random sampling proportional to size. Confirmatory factor analyses were employed to assess the factor structure and construct validity of Amharic version of the DASS-21. Cronbach alpha coefficient and corrected item total correlation was calculated to assess the internal consistency of Amharic version of DASS-21.

**Result:**

Among 435 undergraduate students who participated in the study, 246(56.6%) were the age of 18–25 year and majority 347(79.8%) were males. Regarding their year of study; 200 (46.0%) were first year students. Confirmatory factor analysis indicates a good model fit of the three correlated factors (Comparative fit index (CFI) = 0.92 with root mean square error of approximation (RMSEA) = 0.059[0.052–0.066] and standardize root mean residual SRMR = 0.045). The internal consistency of overall DASS-21 and each sub scale were in acceptable range (0.91, 0.82, 0.77 and 0.77) respectively.

**Conclusion:**

Amharic version of DASS-21 was found to be a valid and reliable instrument to measure the mental problem especially, Depression, Anxiety and Stress among university students.

## Background

The world health organization described mental health as fundamental to human health. According to reports mental health problems are the first cause of disability and a major public health issue worldwide due to progression, difficulties in therapeutic management and increasing prevalence [1]. 

As it is known depression, anxiety and stress are important indictors for mental health which, if untreated, can have a negative effect on individuals. For early treatment and prevention, it is important to conduct screening, which needs valid and reliable tool to measure in clinical and non-clinical setting [2].

The Depression Anxiety and Stress Scale (DASS) is a commonly used self-report questionnaire to measure symptoms of depression, anxiety, and stress. It is designed to provide a comprehensive assessment of these psychological constructs in individuals. When using the DASS for research or clinical purposes, it is essential to consider the validity and reliability of the scale [3]. Since its introduction in 1995, the original version DASS-42 and its short form DASS-21 have been widely used to assess depression, anxiety and stress among different population [4].

Validity refers to the extent to which test measures what it is intended to measure [5]. In the case of the DASS, studies have found strong evidence of its validity. Numerous studies have shown that the DASS has good convergent validity, meaning it correlates strongly with other established measures of depression, anxiety, and stress. Additionally, the DASS demonstrates good discriminant validity, as it shows a distinctive pattern of scores for each construct [6–8]. This indicates that the scale is measuring distinct and separate constructs of depression, anxiety, and stress.

The DASS-21 has been translated into over 30 languages, including Vietnamese, Hindi, Polish, and Persian [9–13]. Confirmatory factor analysis was conducted to validate the translated version both in clinical and non-clinical samples. In line with the original study which propose a three-factor model, most translated study revealed the validity and reliability of the three-factor model [14–16]. But depending on background information and type of population in which the test was employed, a number of literatures results were found to be inconsistent when observed among adults ranging between one to four factors [3, 17, 18]. 

Reliability refers to the consistency and stability of the scale’s measurement across different situations and times [19]. The DASS has been found to have good internal consistency, as demonstrated by high Cronbach’s alpha coefficients for all three subscales (depression, anxiety, and stress), Cronbach’s alpha coefficients, which range from 0.83 to 0.94 for depression, 0.76 to 0.87 for anxiety, and 0.79 to 0.91 for stress. This result was supported by subsequent studies conducted in both clinical and non-clinical samples [6, 10, 20]. This suggests that the items within each subscale are strongly related to each other, indicating that the scale is measuring the intended construct.

According to previously conducted research, DASS have demonstrated a consistent result about its psychometric property among university students. It has been shown to be reliable and valid with three-factor structure [16, 21, 22].

In recent years, researchers from Ethiopia have used the DASS in their studies on different samples [23, 24]. To date, none of the published articles that adopted the DASS-21, tested the validity and reliability of the translated version. Therefore, this study aimed to test the validity and reliability of the DASS- 21 Amharic version.

## Method

### Study design, study period and participants

An institutional based cross-sectional study was conducted using a self-administered questionnaire. The students were asked to complete a set of questions consisting of two parts: demographic information and the DASS-21. The study was conducted at Ethiopian Defense University College of Health Science. Undergraduate students were recruited from college of health science.

A total of 435 students from different departments in accordance with their proportional size were participated in this study and all respective departments were included. As to the sampling technique, the study units were selected from each department and year of study using simple random sampling proportional to size.

### Data collection

Two trained academic staff distributed the Amharic version of the DASS-21 instrument along with a consent form to students before administering the questioner. The students were given a copy of the written instructions and objectives of the study. Informed consent had been taken from all the participants. The participants were assured of the confidentiality of the information provided and had an option of refusal to participate in the survey. The anonymous questionnaire was distributed amongst students during breaks from their teaching schedule and the researchers were collect the completed questionnaires. The questionnaires were taken 20–30 min.

### Instrument

The English version of the DASS-21 questionnaire was translated into Amharic by using standard guidelines, including independent forward and back translation by using language experts.

This 21-item questionnaire contains three subscales including depression (seven items), anxiety (seven items), and stress (seven items). The students responded to the items on a 4-point Likert scale (0 = never a problem, 1 = sometimes a problem, 2 = often a problem, and 3 = almost always a problem). According to the DASS-21 scoring algorithm, higher scores indicated higher depression, anxiety and stress. Total score is calculated by summing the scores for each subscale. Moreover, DASS scoring manual have provided cut-off scores for defining normal (0–4 for depression, 0–3 for anxiety and 0–7 for stress), mild (5–6 for depression, 4–5 for anxiety and 8–9 for stress), moderate (7–10 for depression, 6–7 for anxiety and 10–12 for stress), severe (11–13 for depression, 8–9 for anxiety and 13–14 for stress) and extremely severe (> 14 for depression, > 10 for anxiety, > 17 for stress) scores [25].

Two bilingual psychologists translated the DASS-21 from English to Amharic, without knowing the original wording of the DASS-21. The translated versions were compared item-by-item, and minor discrepancies were resolved by consensus. The Amharic DASS-21 was then piloted with 30 undergraduate students from a private medical college. Adjustments to the translation were made based on the pilot results. Ethical clearance was obtained from the Research Ethics Committee of Ethiopian Defense University college health science.

### Statistical analysis

The reliability of the DASS-21 was examined by Cronbach’s alpha coefficient. A coefficient equal to or greater than 0.7 was considered to be a satisfactory level of reliability. Corrected Item total correlation also computed [19].

In order to evaluate the construct validity of the scale, confirmatory factor analysis (CFA) was used. Generally, CFA investigates the relationship between a set of observed variables (the items of the DASS-21) and a set of latent constructs (depression, anxiety, and stress subscales). In the present study, we investigated whether or not the hypothesized three-factor model fit the data well for the sample. Several criteria were used to assess the goodness of fit of the model, including chi-square statistics, root mean square error of approximation (RMSEA), Tuker-Lewise index (TLI), comparative fit index (CFI) and Standardize root mean residual (SRMR). Since chi-square statistics are known to be sensitive to large samples, this test may not be a realistic fit index, and therefore, the other above-mentioned fit indices were considered for assessing goodness of fit of the model. Values of CFI and TLI ≥ 0.90, and RMSEA ≤ 0.08 can support acceptable model fit. Stata version 18 were used to conduct CFA [5, 26].

### Result

#### Demographic characteristics of respondents

Out of the 435 undergraduate students who participated in the study, 246 (56.6%) were between the ages of 18 and 25, and the majority, 347 (79.8%), were male. In terms of their year of study, 200 (46.0%) were first-year students, 74 (17.0%) were in their second year, 107 (24.6%) were in their third year, 52 (12.0%) were in their fourth year, and 2 (0.5%) were in their fifth year. Overall mean Depression, Anxiety and Stress score of the whole study participants were 4.7 ± 3.8[CI: 4.3–5.1], 3.5 ± 3.3[CI: 3.1–3.8], 4.6 ± 3.8[CI: 4.2–4.9] respectively.

### Internal consistency of Amharic of DASS-21

The Cronbach’s alpha coefficient was 0.91for the total score of the Amharic version of DASS-21; it was 0.82 for depression, 0.77 for anxiety, and 0.77 for stress, indicating acceptable internal consistency of the scale [27]. The corrected item-total correlations for the DASS-21 were ranged from 0.51 to 0.70 and in an acceptable range (Table 1).

**Table 1:** Reliability and corrected item-total correlation for DASS-21 items and subscales (*n* = 435)

Items	Corrected Item-Total correlation	Cronbach Alpha
**DAS-Depression**		
I found it difficult to work up the initiative	0.7	0.79
I felt that I had nothing to look forward to	0.6	0.81
I felt down-hearted and blue	0.7	0.80
I was unable to become enthusiastic about anything	0.7	0.79
I felt I was not worth much as a person	0.7	0.79
I felt that life was meaningless	0.6	0.80
I could not seem to experience any positive feeling	0.7	0.79
Overall		**0.82**
**DAS-Anxiety**		
I was aware of dryness of my mouth	0.6	0.74
I experienced breathing difficulty	0.6	0.74
I experienced trembling	0.5	0.75
I was worried about situations in which I might panic and make a fool of myself	0.7	0.73
I felt I was close to panic	0.6	0.74
I was aware of the action of my heart in the absence of physical exertion	0.6	0.75
I felt scared without any good reason	0.7	0.73
Overall		**0.77**
**DAS-Stress**		
I found it hard to wind down	0.6	0.74
I tended to over-react to situations	0.4	0.78
I felt I was using a lot of nervous energy	0.6	0.75
I found myself getting agitated	0.7	0.72
I found it difficult to relax	0.7	0.72
I was intolerant of anything that kept from getting on with what I was doing	0.6	0.73
I felt that I was rather touchy	0.6	0.76
**Overall**		**0.77**

### Construct validity of Amharic of DASS-21

**Fig. 1:**
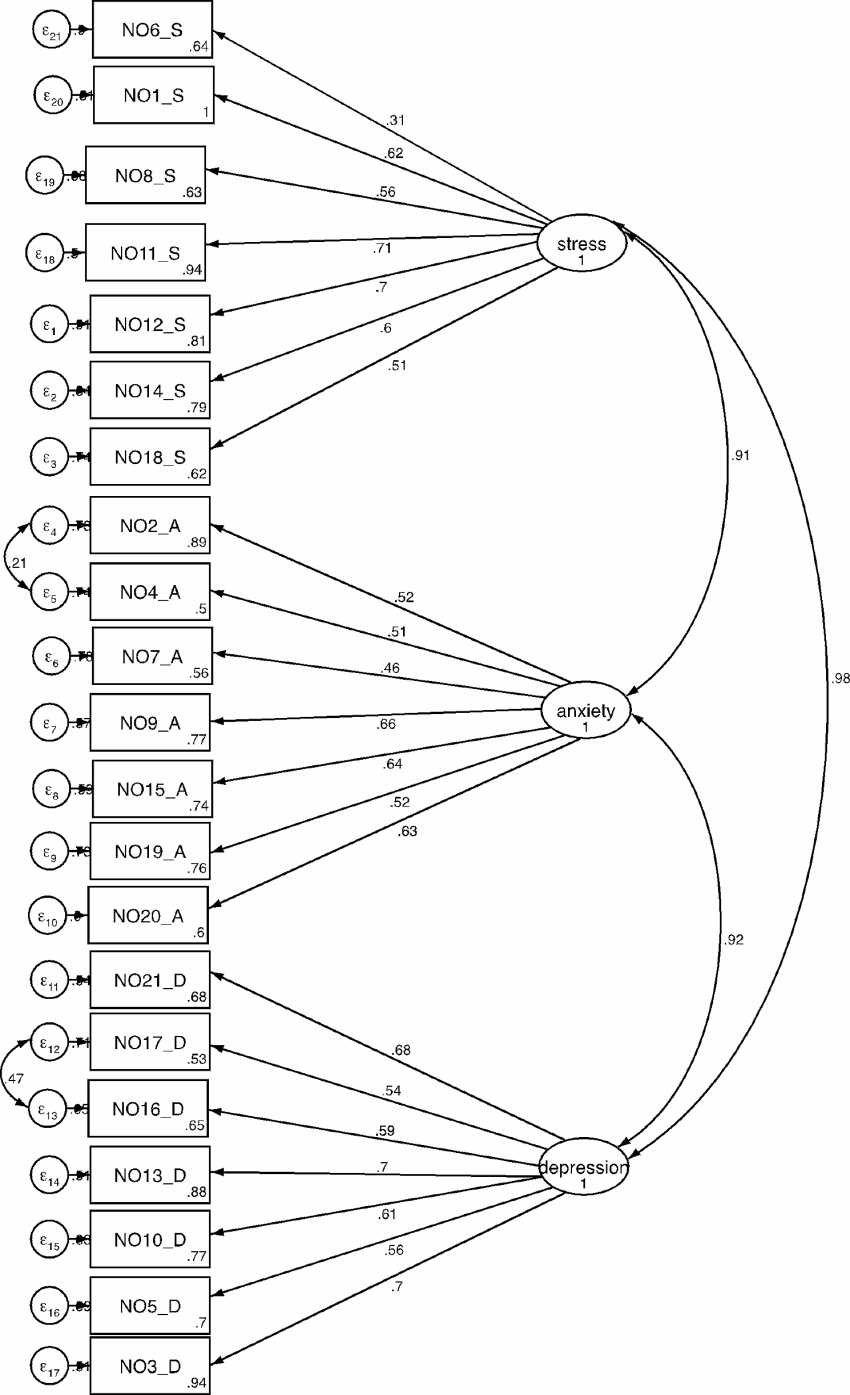
Standardize factor loading of Amharic version of DASS-21

The CFA was used to evaluate the goodness of fit of the three-factor models of Amharic version of DASS-21. The analysis yielded a 21-item three-factor model that fit the data of Amharic version of DASS-21 (Fig. 1) The CFA revealed the acceptable fit indexes of the three-factor model. The goodness-of-fit indices showed, RMSEA 0.059, TLI 0.91, CFI 0.92 and SMRS of 0.045 (Table 2). The factor loadings ranged from 0.46 to 0.71 for all items except one item which was 0.31 (Table 3). Accordingly, the goodness of fit of the three-factor model was established, suggesting the stability of this instrument.

**Table 2:** Fit indices of confirmatory factor analysis for Amharic version DASS-21

Model	X^2^ (df)	CFI	RMSEA(95%CI)	TLI	SRMR
Three factors model with correlated error	445.5(184)	0.92	0.059(0.052–0.066)	0.91	0.046

***RMSEA: root mean square error of approximation CFI: Comparative fit index SRMR: standardize root mean residual***

**Table 3:** Confirmatory factor analysis of the three-factor model of Amharic version DASS-21

Items	Factor loading
**DASS-Depression**	
I found it difficult to work up the initiative	0.68
I felt that I had nothing to look forward to	0.54
I felt down-hearted and blue	0.59
I was unable to become enthusiastic about anything	0.70
I felt I was not worth much as a person	0.61
I felt that life was meaningless	0.56
I could not seem to experience any positive feeling	0.70
**DASS-Anxiety**	
I was aware of dryness of my mouth	0.52
I experienced breathing difficulty	0.51
I experienced trembling	0.46
I was worried about situations in which I might panic and make a fool of myself	0.66
I felt I was close to panic	0.64
I was aware of the action of my heart in the absence of physical exertion	0.52
I felt scared without any good reason	0.63
**DASS-Stress**	
I found it hard to wind down	0.31
I tended to over-react to situations	0.62
I felt I was using a lot of nervous energy	0.56
I found myself getting agitated	0.71
I found it difficult to relax	0.70
I was intolerant of anything that kept from getting on with what I was doing	0.60
I felt that I was rather touchy	0.51

### Correlation between the subscales of Amharic version of DASS-21

The subscales of Amharic version of DASS-21 were found to be significantly correlated. The coefficients for the correlations of subscales were, 0.92 for depression and anxiety, 0.98 for depression and stress, and 0.92 for anxiety and stress.

## Discussion

The aim of this study was to assess the construct validity and reliability of the Amharic version of the DASS-21 among health science students. It is known that the DASS-21 has been translated into various languages and used in different cultural contexts [16, 28, 29]. As to the knowledge of the author is concerned, Amharic version of this scale has not been validated among students but the instrument was used widely for research.

The study findings support the three-factor model as also reported in previous studies and as the original study proposed. the standardized factor loading ranged from 0.46 to 0.71, only one item factor loading was 0.31 otherwise, the study identified the same factor structure and pattern with the tripartite model of the DASS-21 and support the theoretical perspective for the three dimensions of scale [4, 18]. 

In order to test the construct validity of the Amharic version of DASS-21, we have conducted confirmatory factor analysis using Stata version 18. We have tested the three-factor model. fit indexes of the CFA model showed that the three-factor structure of DASS-21 was a good fit for the present data. this result was in line with the original three-factor model and also it is consistent with previous reported validation studies in different language and population segment [4, 12, 15, 30]. 

As far as factor structure of DASS-21 concerned disagreement of results was observed. This may be due to cultural difference and the population segment involved in study. A study conducted among Korean university students and among adolescent in North Macedonia, in both studies result demonstrated a bi-factor model [17, 18], Another study conducted among a sample of Vietnamese adolescents, the data supported a four-factor model [20].

We have examined the internal consistency of Amharic version of DASS-21 using Cronbach alpha. In the original study, the internal reliability of the DASS-21 has been established through the examination of Cronbach’s alpha coefficients, which range from 0.83 to 0.94 for depression, 0.76 to 0.87 for anxiety, and 0.79 to 0.91 for stress [4]. In line with the original study, our finding demonstrated acceptable internal consistency reliability of the total scale and each sub-scale, with Cronbach alpha coefficient of 0.91for total scale, 0.82 for depression, 0.77 for anxiety and 0.77 for stress. Similarly, the internal consistency of this scale was good, as evidenced by the corrected item –total correlation for the entire scale ranging from 0.4 to 0.7. The results also in line with other previously conducted validation studies of DASS-21 [28, 31, 32]. accordingly, the result suggest that we can use three scales measured in DASS-21 separately or as a combined form in order to assess symptoms of depression, anxiety and stress.

### Strength and limitation

The strength of this study is that it is the first study which examine the validity and reliability of Amharic version of DASS-21 in a sample of health science students in Ethiopia. There are some limitations in this study. Firstly, the study was cross sectional and the data can’t show test-retest reliability overtime. Secondly the study only tests the construct validity. finally, only undergraduate health sciences students were involved in the study which compromised generalization.

## Conclusion

In summary, the results from this study provide evidence that the Amharic version DASS-21 is valid and reliable and suitable for use as a screening tool for symptoms of mental health problems, Depression, Anxiety and Stress among University students.

As the DASS-21 has now been translated to more than 30 languages, the use of Amharic version DASS-21 will provide additional opportunities for cross-cultural comparison. However, to further support the validity and reliability of DASS-21, additional study is recommended.

## Data Availability

Pertinent data are available in this manuscript. Additional data can be requested from the corresponding author upon reasonable request.

## Abbreviations

DASS-21: Depression Anxiety Stress Scale
EDU: Ethiopian Defense University
EFA: Exploratory Factor Analysis
CFA: Confirmatory Factor Analysis
RMSEA: Root mean square error of approximation
SRMR: Standardize root mean residual
CFI: Comparative fit index
